# Development of interstitial pneumonia during treatment with eribulin: a case report

**DOI:** 10.1186/s13104-017-2882-4

**Published:** 2017-11-06

**Authors:** Kota Nakamura, Motoyasu Kato, Yosuke Miyashita, Osamu Nagashima, Shinichi Sasaki, Shigeru Tominaga, Kazuhisa Takahashi

**Affiliations:** 1 Department of Respiratory Medicine, Juntendo University Urayasu Hospital, 2-1-1, Tomioka, Urayasu, Chiba 273-0021 Japan; 2 Department of Respiratory Medicine, Juntendo University Graduate School of Medicine, 3-1-3, Hongo, Bunkyo-ku, Tokyo, 113-8431 Japan

**Keywords:** Eribulin, Drug-induced lung toxicity, Drug-induced interstitial pneumonia, Interstitial pneumonia, Organized pneumonia

## Abstract

**Background:**

Eribulin is typically used to treat patients with advanced breast cancer, and anti-cancer agents often cause the development of interstitial pneumonia in Japanese patients with advanced cancer. However, few case reports have addressed eribulin-induced interstitial pneumonia. Herein, we report a rare case of interstitial pneumonia—specifically, organized pneumonia—during treatment with eribulin in a patient with advanced breast cancer.

**Case presentation:**

A 52-year-old Japanese woman was diagnosed as having advanced breast cancer 3 years before the admission described in the present report. She had received eribulin as third-line chemotherapy. Five days after her second treatment with eribulin, she was admitted to our hospital with dyspnea and dry cough. Upon admission, a chest computed tomography scan showed consolidation, with air bronchograms along the bronchovascular bundle of both lower lobes. The patient’s serum levels of sialylated carbohydrate antigen Krebs von den Lungen-6 were high, as were her surfactant protein-D levels. There was no evidence of heart failure, renal failure, or infection. Based on the clinical cause, as well as on the findings of organized pneumonia, the patient was diagnosed as having interstitial pneumonia and treated with corticosteroids. After the initiation of steroid treatment, her respiratory condition and chest radiological findings improved.

**Conclusions:**

This case reveals an association between eribulin treatment and interstitial pneumonia. To our knowledge, this is the first case report to describe eribulin-induced organized pneumonia. Clinicians should be aware that interstitial pneumonia can develop during treatment with anti-cancer agents.

## Background

Eribulin mesylate is used to treat patients with advanced breast cancer (BC). Phase III trials involving patients with heavily pre-treated metastatic BC demonstrated that the overall survival time of patients treated with eribulin is significantly longer than that of patients treated with other cytotoxic therapies [1]. However, a phase II study showed that the incidence of interstitial pneumonia (IP) during eribulin treatment is approximately 1.2% in Japanese patients with advanced BC [2]. Despite this, few case reports have described the development of eribulin-induced IP. In the present report, we present a rare case of IP in a patient with advanced BC who was undergoing eribulin treatment.

## Case presentation

A 52-year-old Japanese woman was diagnosed as having advanced BC with a sensitive estrogen receptor and without human epidermal growth factor receptor 2. This diagnosis took place about 3 years prior to the admission described in the present report. She received four courses of combination therapy with epirubicin, cyclophosphamide, and fluorouracil (ECF) for 6 months, and then eight courses of docetaxel (DTX) for 10 months. After these two regimens, a malignant pleural effusion developed; therefore, the disease was considered progressive. After DTX treatment, the patient received two estrogen receptor inhibitors, tamoxifen for 6 months, fulvestrant for 4 months, and an aromatase inhibitor (letrozole) for 11 months. Despite this, the patient’s BC metastasized to the liver; the attending physician therefore considered the disease progressive and began treatment with eribulin. Five days after she began her second treatment with eribulin, she was admitted to our hospital for dyspnea.

Upon admission, her initial vital signs included a body temperature of 37.5 °C and an oxygen saturation of 88% in room air. A physical examination revealed fine crackles in both lower lung fields. Her laboratory test results were as follows: white blood cells—1200 cells/µL (neutrophils—600 cells/µL); serum lactate dehydrogenase (LDH)—749 IU/L (normal: 119–229 IU/L); serum sialylated carbohydrate antigen Krebs von den Lungen-6 (KL-6) level—3782 U/mL (normal: < 500 U/mL); serum surfactant protein-D (SP-D)—158 ng/mL (normal: < 110 ng/mL); serum C-reactive protein (CRP)—3.9 mg/dL (normal: < 0.3 mg/dL); and plasma (1-3)-beta-d-glucan—12 pg/mL (normal: < 20 pg/mL). The patient was negative for serum antibodies associated with connective tissue diseases. Furthermore, her sputum, urine, and blood cultures yielded no microbial growth, and she was negative for a rapid flu test, cytomegalovirus antigen, *Mycoplasma* antigen in the pharynx, and *Streptococcus pneumoniae* and *Legionella* antigen in the urine.

**Fig. 1:**
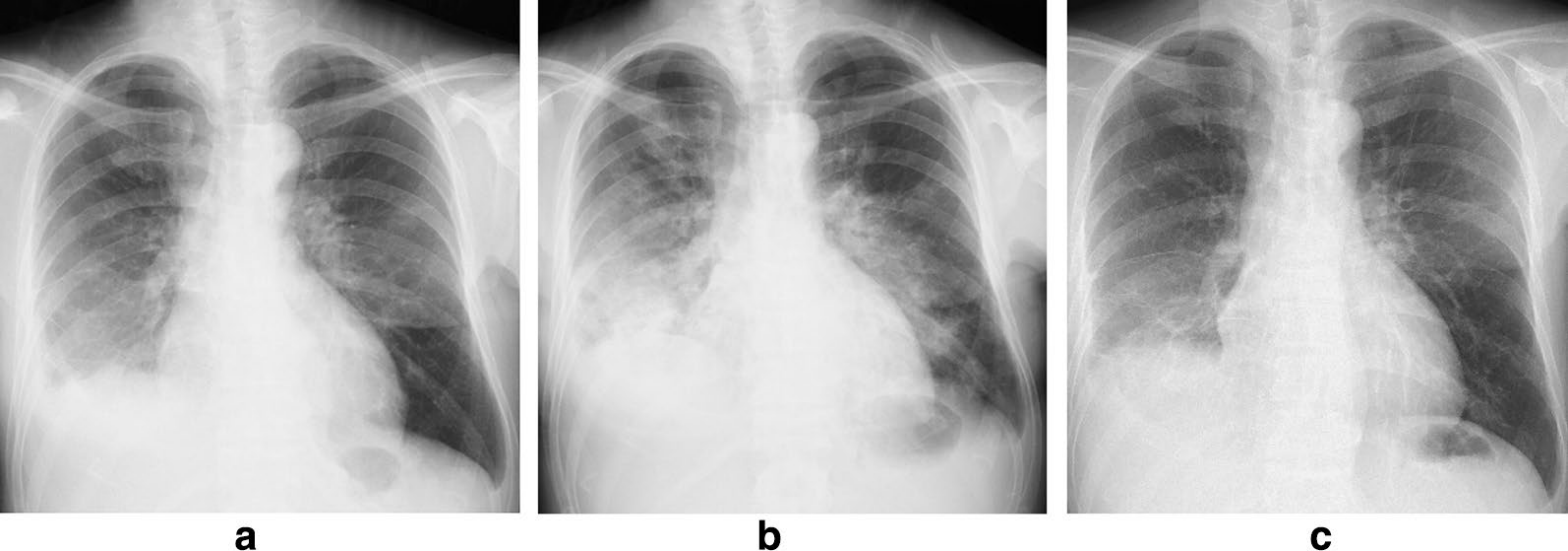
Chest radiological findings. Chest radiograph findings showing **a** no abnormal shadow before admission, **b** a consolidation in the right lower lung field upon admission, and **c** an improvement of the consolidation after treatment with prednisolone

**Fig. 2:**
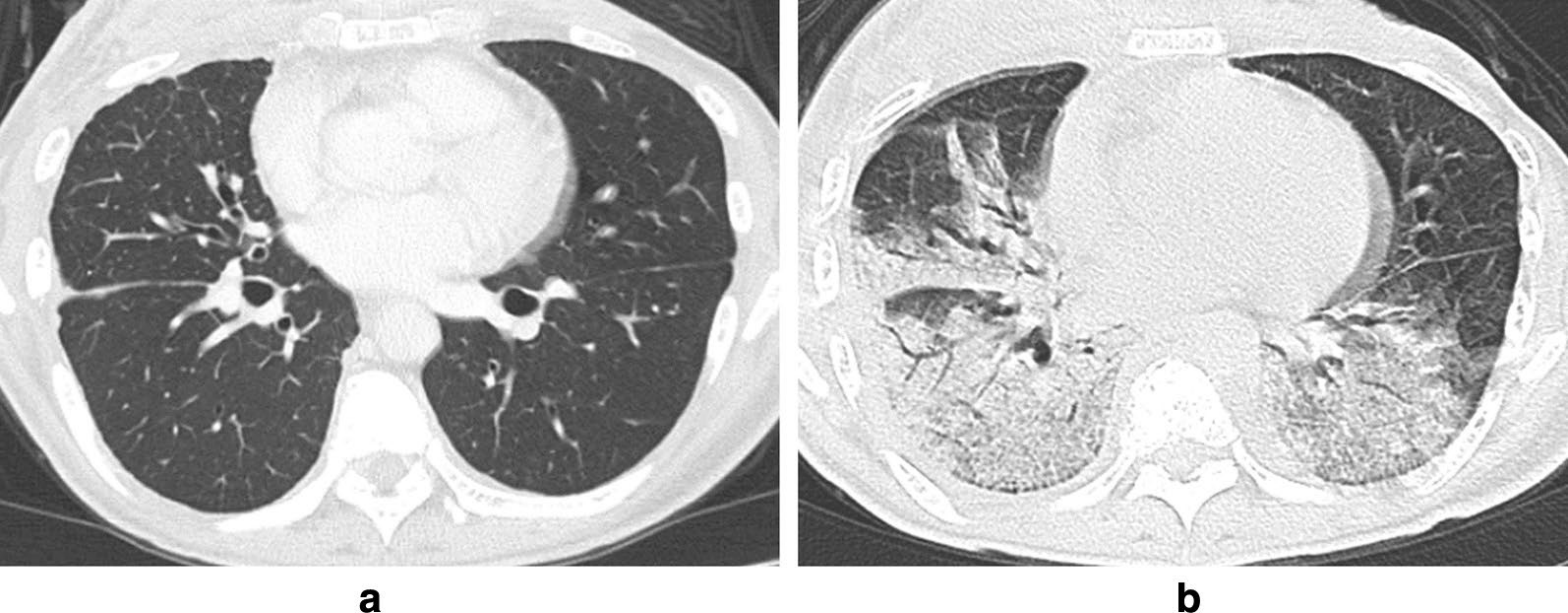
Findings on chest high-resolution computed tomography. A high-resolution computed tomography scan of the chest showing **a** no abnormal shadow prior to admission and **b** a consolidation along the bronchovascular bundle in both lower lung lobes upon admission

A chest radiograph (Fig. 1b) showed consolidation in the right lower lung field, and a chest high-resolution computed tomography (HRCT) scan showed consolidation with air bronchograms along the bronchovascular bundles in both lower lobes (Fig. 2b). Prior to the admission described in the present study, the patient had no pre-existing interstitial shadow on her chest radiograph (Fig. 1a) and HRCT (Fig. 2a). Moreover, she showed no evidence of heart or renal failure; that is, her brain natriuretic peptide and creatinine levels were within the normal range.

A year before the admission described in the present report, the patient was prescribed brotizolam, tramadol, loxoprofen, and famotidine to treat insomnia, chronic gastritis, and carcinomatous pain. Upon admission, we treated her with the antibiotic cefepime for 5 days, as well as with carbocysteine and dextromethorphan hydrobromide hydrate for sputum and cough. However, her respiratory symptoms and radiological findings were not improved by this treatment. Five days after her admission, we performed a bronchoalveolar lavage (BAL) in the right B^4^ bronchus. The total cell count, lymphocyte, neutrophil, eosinophil, and macrophage percentages, and CD4/CD8 ratio values in the BAL fluid (BALF) were 10.8 × 10^5^ cells/mL (normal range: 0.7–2.0 × 10^5^ cells/mL), 13, 2, 0, 85%, and 0.63 (normal range: 1.5–3.2), respectively. The polymerase chain reaction (PCR) assay of the BALF for *Pneumocystis jirovecii* was negative.

Based on these findings, including the clinical course and chest HRCT, we concluded that the patient had developed IP, specifically organized pneumonia (OP), and we therefore initiated treatment with prednisolone (1 mg/kg/day), gradually decreasing the dosage every 2 weeks. The patient’s respiratory condition and chest radiograph findings (Fig. 1c) had improved after 2 weeks of prednisolone treatment.

## Discussion

Eribulin is known as a microtubule polymerization-inhibiting compound (MPIC) [3]. Such compounds are often used to treat patients with advanced BC and non-small cell lung cancer (NSCLC). More specifically, eribulin is classified as a non-taxane MPIC and is similar to vinorelbine (VNR). When choosing an MPIC to treat patients with advanced NSCLC, it may be advisable to use paclitaxel (PTX) in patients with IP, as the drug’s associated incidence of acute IP exacerbation is below 1%, as shown by a prospective trial in Japan [4]. In contrast, the incidence of IP acute exacerbation is 15.8% in patients receiving VNR plus cisplatin treatment as a first-line therapy [5]. The incidence of IP is 4.6% in patients with advanced NSCLC treated with DTX [6]. Even though BC differs from NSCLC, it may be that MPICs are associated with IP development in patients with any cancer.

We evaluated the chest HRCT pattern and BALF to determine the IP pattern. The chest HRCT findings had the characteristics of OP. In the BALF, the total cell count was elevated and the lymphocyte count was slightly elevated. We considered performing a transbronchial lung biopsy to classify the IP pattern; however, we could not do so after the BAL, because the patient’s respiratory condition had worsened.

On a different note, the patient in the present report was negative for the drug lymphocyte stimulation test (DLST) for eribulin in peripheral blood. A positive DLST is considered significant evidence for drug-induced IP in Japan; however, the sensitivity and specificity of the DLST for anti-cancer agents is yet unknown [7]. As infections, heart failure, and renal failure had been excluded by the appropriate tests, we clinically diagnosed the patient as having eribulin-related OP [8].

In the present report, our patient received many anti-cancer regimens, including ECF, DTX, two estrogen receptor inhibitors, and an aromatase inhibitor before treatment with eribulin. The last both ECF and DTX treatments were completed over 6 months before the onset of OP. DTX-induced IP has only ever been reported in a Japanese NSCLC patient. Specifically, IP developed four times during DTX treatment. In the present report, just before eribulin treatment, the patient received two estrogen-receptor inhibitors and an aromatase inhibitor. The incidence of IP during treatment with these agents was below 0.1% in a Japanese post-marketing surveillance. For this reason, we did not suspect that the agents used prior to eribulin had led to IP development.

Over 1 year before the admission described in the current report, the patient had begun using many medicines, including brotizolam, tramadol, loxoprofen, and famotidine for insomnia, chronic gastritis, and carcinomatous pain. These medicines were not suspected to have caused the IP, because the treatment had been long-term. During the admission described in the present report, the patient also received carbocysteine and dextromethorphan hydrobromide hydrate to treat sputum and cough, as well as cefepime. We started to use these medicines after the patient’s IP had developed, so they were not suspected to have caused her IP either. For the same reason, we presumed that none of the other medicines that the patient began to use during the present admission were associated with IP development.

Although many reports have addressed drug-induced IP, only one case report has focused on IP development during eribulin treatment. Specifically, Ishida et al. described eribulin-induced IP in a patient with recurrent BC; the patient’s chest HRCT demonstrated bilateral ground glass opacity, with thickening of the interlobular and intralobular septal lines [9]. However, the chest HRCT findings differed in the present study; in fact, because so few reports have addressed eribulin-induced IP development, it was difficult for us to investigate the HRCT features of the disease. Therefore, investigators must conduct further studies and reports to consider the incidence, risk factors, and radiological or pathological features of eribulin-induced IP.

## Conclusion

To the best of our knowledge, this is the first case of OP development during treatment with eribulin. Clinicians should be aware that IP can develop during anti-cancer treatment.

## Abbreviations

BAL: bronchoalveolar lavage
BALF: bronchoalveolar lavage fluid
BC: breast cancer
CRP: C-reactive protein
DLST: drug lymphocyte stimulation test
DTX: docetaxel
ECF: epirubicin, cyclophosphamide, and fluorouracil
HRCT: high-resolution computed tomography
IP: interstitial pneumonia
KL-6: sialylated carbohydrate antigen Krebs von den Lungen-6
LDH: lactate dehydrogenase
MPIC: microtubule polymerization-inhibiting compound
NSCLC: non-small cell lung cancer
OP: organized pneumonia
PCR: polymerase chain reaction
PTX: paclitaxel
SP-D: surfactant protein-D
VNR: vinorelbine

## References

[1] Cortes J, O’Shaughnessy J, Loesch D, Blum JL, Vahdat LT, Petrakova K, Chollet P, Manikas A, Diéras V, Delozier T, Vladimirov V, Cardoso F, Koh H, Bougnoux P, Dutcus CE, Seegobin S, Mir D, Meneses N, Wanders J, Twelves C, EMBRACE (Eisai Metastatic Breast Cancer Study Assessing Physician’s Choice Versus E7389) investigators (2011). Eribulin monotherapy versus treatment of physician’s choice in patients with metastatic breast cancer (EMBRACE): a phase 3 open-label randomised study. Lancet.

[2] Aogi K, Iwata H, Masuda N, Mukai H, Yoshida M, Rai Y, Taguchi K, Sasaki Y, Takashima S (2012). A phase II study of eribulin in Japanese patients with heavily pretreated metastatic breast cancer. Ann Oncol.

[3] Jordan MA, Kamath K, Manna T, Okouneva T, Miller HP, Davis C, Littlefield BA, Wilson L (2005). The primary antimitotic mechanism of action of the synthetic halichondrin E7389 is suppression of microtubule growth. Mol Cancer Ther.

[4] Minegishi Y, Sudoh J, Kuribayasi H, Mizutani H, Seike M, Azuma A, Yoshimura A, Kudoh S, Gemma A (2011). The safety and efficacy of weekly paclitaxel in combination with carboplatin for advanced non-small cell lung cancer with idiopathic interstitial pneumonias. Lung Cancer.

[5] Okuda K, Hirose T, Oki Y, Murata Y, Kusumoto S, Sugiyama T, Ishida H, Shirai T, Nakashima M, Yamaoka T, Ohnishi T, Ohmori T (2012). Evaluation of the safety and efficacy of combination chemotherapy with vinorelbine and platinum agents for patients with non-small cell lung cancer with interstitial lung disease. Anticancer Res.

[6] Kenmotsu H, Naito T, Kimura M, Ono A, Shukuya T, Nakamura Y, Tsuya A, Kaira K, Murakami H, Takahashi T, Endo M, Yamamoto N (2011). The risk of cytotoxic chemotherapy-related exacerbation of interstitial lung disease with lung cancer. J Thorac Oncol.

[7] Matsuno O, Okubo T, Hiroshige S, Takenaka R, Ono E, Ueno T, Nureki S, Ando M, Miyazaki E, Kumamoto T (2007). Drug-induced lymphocyte stimulation test is not useful for the diagnosis of drug-induced pneumonia. Tohoku J Exp Med.

[8] Matsuno O (2012). Drug-induced interstitial lung disease: mechanisms and best diagnostic approaches. Respir Res.

[9] Ishida H, Homma T, Ishida K, Sugiyama T, Kusumoto S, Shirai T, Nakashima M, Ohnishi T, Hirose T (2013). Pneumonia induced by eribulin mesylate in a patient with recurrent breast cancer. Int Cancer Conf J.

